# Frugal Learning Methods for Kidney Segmentation in Non-Contrast MRI

**DOI:** 10.3390/jcm15145747

**Published:** 2026-07-22

**Authors:** Jan Podlaszewski, Artur Klepaczko, Ludomir Stefańczyk, Marcin Majos

**Affiliations:** 1 Institute of Electronics, Lodz University of Technology, 90-924 Łódź, Poland; artur.klepaczko@p.lodz.pl; 2 Department of Radiology and Diagnostic Imaging, Medical University of Lodz, 90-419 Lodz, Poland; ludomir.stefanczyk@umed.lodz.pl; 3 Department of Normal and Clinical Anatomy, Medical University of Lodz, 90-419 Lodz, Poland; marcin.majos@umed.lodz.pl

**Keywords:** kidney segmentation, annotation scarcity, semi-supervised learning, weak labeling, transfer learning, data augmentation, non-contrast MRI

## Abstract

**Background/Objectives**: Chronic kidney disease is a growing global health concern, necessitating effective tools for early detection and monitoring. While non-contrast T1-weighted magnetic resonance imaging offers a non-invasive means to assess kidney morphology, robust automated segmentation remains challenging due to limited annotated data, high inter-patient variability, and low signal-to-noise ratios. **Methods**: In this study, we address these obstacles by developing and evaluating a series of frugal learning methodologies for kidney segmentation in non-contrast MRI. Building upon the U-Net architecture, we aim to maximize segmentation accuracy despite scarce labeled data. Our experimental framework leverages three diverse datasets to evaluate performance-boosting strategies such as transfer learning: a clinically relevant local cohort (the Barlicki dataset) as the primary target domain and two auxiliary public datasets (AMOS22 and AbdomenCT). Utilizing these data streams, we systematically compare seven frugal learning strategies incorporating data augmentation, semi-supervised learning, and weak supervision against a fully supervised baseline. **Results**: The results demonstrate that frugal learning methods enable accurate and reliable kidney segmentation while substantially reducing the need for manual annotations. The best-performing semi-supervised and transfer learning approaches achieved a Dice similarity coefficient of 0.89, which was only moderately lower than that of the fully supervised model (Dice = 0.92). **Conclusions**: This work highlights the potential of data-efficient deep learning techniques to accelerate the adoption of automated kidney segmentation in clinical workflows, particularly in settings where annotated medical images are limited.

## 1. Introduction

Kidneys play a fundamental role in maintaining homeostasis through blood filtration, electrolyte regulation, and blood pressure control. Chronic kidney disease (CKD) affects approximately 10–15% of the adult population and frequently remains undiagnosed until the advanced stages, making early detection and monitoring essential [1]. Non-invasive imaging techniques, particularly MRI, are increasingly used for renal assessment because they provide kidney-specific structural information that is unavailable from routine blood biomarkers alone [2,3,4,5]. In this study, we focus on T1-weighted non-contrast MRI, which enables evaluation of kidney morphology, including size, volume, and cortical thickness, as indicators of structural damage and disease progression.

Quantitative assessment of kidney morphology requires accurate organ delineation, which is typically a tedious task when performed manually. Consequently, numerous studies have focused on developing automated kidney segmentation techniques, thus relieving the workload imposed on radiologists. However, the literature review (see Section 2 and references therein) indicates that most of these methods target contrast-enhanced CT or MRI images. While these modalities offer superior resolution and signal-to-noise ratios, they are unsuitable for patients with CKD due to compromised renal filtration of contrast agents. On the other hand, approaches tailored to T1-weighted non-contrast MRI, particularly using Dixon-VIBE acquisitions, remain limited. This setting is inherently more challenging due to lower signal-to-noise ratios, reduced tissue contrast, and substantial inter-patient variability. Furthermore, the development of robust deep learning models is hindered by the scarcity of annotated datasets, as manual delineation of kidneys in MRI is time-consuming and expertise-intensive.

To address these limitations, we propose a systematic and data-efficient framework for kidney segmentation in non-contrast T1-weighted Dixon MRI explicitly designed for low-annotation regimes. Unlike prior work, which typically evaluated a single training paradigm, we conduct a comprehensive and controlled comparison of multiple frugal learning strategies within a unified experimental setting. The key scientific contributions of this work are as follows:

- Systematic evaluation of diverse frugal learning strategies for kidney segmentation in non-contrast Dixon-VIBE MRI under identical conditions, including transfer learning (CT- and MRI-based), data augmentation with cross-domain datasets, semi-supervised learning, weak supervision using Grad-CAM-derived pseudo-labels, and self-supervised autoencoder pretraining, all under identical conditions. This enables the fair and methodologically consistent comparison that is currently missing in the literature.
- Quantitative analysis of segmentation performance across extreme low-data regimes ranging from 100 training slices to the full dataset, demonstrating how different frugal learning strategies behave when labeled data are severely constrained.
- Demonstration that frugal learning can match or exceed fully supervised baselines with limited annotations. Our results show that semi-supervised learning achieves the highest performance (Dice ≈0.96 on validation), while autoencoder pretraining and weak supervision offer strong alternatives in ultra-low-data settings. These findings highlight that label-efficient training can substantially reduce annotation requirements without sacrificing accuracy.

Collectively, these contributions advance the state of the art by shifting the focus from purely accuracy-driven segmentation toward data-efficient solutions tailored to realistic imaging constraints. To the best of our knowledge, this is the first study to provide such a controlled analysis of frugal learning strategies for kidney segmentation in non-contrast MRI data from CKD patients.

The paper is structured as follows: First, we present the outcomes of a literature review, addressing two key areas: kidney segmentation approaches and frugal learning methods, with particular emphasis on weak supervision and semi-supervision in medical image segmentation. We then describe the experimental methodology and the datasets used in our study. In the Section 4, we compare our proposed frugal learning approaches with a fully supervised model. Finally, we discuss our findings and present conclusions.

## 2. Related Work

### 2.1. Kidney Segmentation

In recent years, deep learning algorithms have revolutionized the field of kidney segmentation, offering significant improvements in segmentation accuracy and efficiency. Among works considering approaches specifically tailored for T1-weighted Dixon-VIBE imaging, the studies [6,7] are particularly noteworthy.

In work [6], a neural network leveraging the U-Net architecture [8] is developed incorporating 3D convolutional layers and residual connections. Separate networks are trained for in-phase, opposed-phase, and water-only images typically acquired through the Dixon method, achieving a Dice similarity coefficient (DSC) of 0.902 and an average total kidney volume deviation of 27 mL. In contrast, Kellner et al. in [7] introduce a 3D convolutional neural network optimized for multi-scale problems and designed to simultaneously perform localization and segmentation tasks using water- and fat-only images as inputs. This approach is validated on a substantial dataset of approximately 10,000 patients. The agreement between segmentation-derived and manually determined kidney volumes ranges from 0.6 to 0.95, as assessed using Spearman and Pearson correlation coefficients. The DSC scores for raw segmentation results are not provided.

Further MRI-oriented research has explored diverse architectures and multimodal strategies. These include Mask R-CNN-based tissue segmentation for functional MRI analysis [9], as well as specialized networks such as FG-CKD-UNet [10], hybrid pipelines [11], multi-scale U-Nets with ASPP blocks [12], and multiparametric models [13]. More recent Transformer-based solutions also address modality variability, enabling training on a mixture of MRI and CT images, even if certain modalities are partially missing [14]. Hybrid approaches have also emerged; for example, ref. [15] integrates U Net–based probability mapping with level-set methods to refine kidney boundaries in Dynamic Contrast-Enhanced (DCE) MRI scans.

The literature based on CT is considerably broader. One group of methods focuses on advanced U-Net variants—2.5D, full 3D, and multi-scale configurations—for kidneys, tumors, and cysts [16,17,18]. Other work incorporates active learning [19,20], boundary-aware feature extraction [21], squeeze-and-excitation attention [22], asymmetric encoder–decoder designs [23], or mathematically inspired architectures such as KANSeg [24]. Hybrid pipelines combining 3D CNNs with deformable registration also appear in the CT domain [25], aiming to better accommodate anatomical variability. Additional studies evaluate segmentation for clinical metrics such as CKD-related renal structural measurements [26].

Beyond MRI and CT, kidney segmentation has also been explored in ultrasound. A notable approach is Fast UNet++ [27], which segments 2D ultrasound slices and extracts anatomical dimensions relevant for disease assessment. Finally, a broad comparison study [28] benchmarks several neural architectures—U-Net [29], ResNet-based U-Net [30], SegNet [31], DeepLabV3+ [32], and Transformer-based designs [33]—on unenhanced abdominal CT, identifying ResNet U-Net as the most effective among the evaluated models.

Across the reviewed literature, MRI-based kidney segmentation methods achieve Dice scores ranging roughly from 0.73 to 0.99, reflecting variation in target structures (whole kidney vs. cortex/medulla) and imaging protocols. CT-based approaches, which constitute the majority of published work, typically report Dice scores in the range of 0.80 to 0.97 for kidney and related structure segmentation, with both 2.5D and full 3D networks demonstrating strong and competitive performance.

Despite the significant progress in kidney segmentation, several limitations can be identified in the existing literature: First, many approaches rely on large, well-annotated datasets, which are often not available in clinical practice, especially for non-contrast MRI. Second, a substantial portion of the reported methods focuses on contrast-enhanced imaging or CT data, where tissue boundaries are more clearly defined. This limits their direct applicability to non-contrast MRI characterized by a lower signal-to-noise ratio and reduced contrast. Finally, more advanced architectures, while achieving high segmentation accuracy, often require increased computational resources and extensive training time, which may hinder their practical deployment. These constraints motivated the exploration of data-efficient (frugal) learning strategies addressed in this work.

### 2.2. Medical Image Segmentation in Annotation-Constrained Scenarios

All the aforementioned studies employ fully supervised learning. Recently, novel methods have emerged aiming to develop segmentation models that address the issue of labeled data scarcity. While significant progress has been made in the context of generic image segmentation, the research on such methods in medical imaging remains limited. In MRI, several works have exploited semi-supervised or synthetic data strategies. Study [34] employs style transfer-based augmentation using CycleGAN-generated [35] multi-phase contrast-enhanced MRI, enabling segmentation with substantially fewer labeled samples. Sharaby et al. [36] also rely on a modified CycleGAN, but focus on learning kidney shape distributions directly from realistic masks, effectively eliminating the need for annotated MRI. In a related direction, Zhang et al. [37] integrate a Pix2Pix augmentation module to synthesize MRI training data. TotalSegmentator [38], in contrast, reduces reliance on large homogeneous datasets by jointly training on MRI and CT through cross-modality learning. However, these approaches often depend on the quality and realism of the generated synthetic data, which may introduce artifacts or distribution shifts affecting downstream segmentation performance. Additionally, cross-modality learning methods can be sensitive to domain discrepancies between imaging modalities, potentially limiting their generalization to specific acquisition protocols.

Several CT-oriented studies likewise have addressed annotation scarcity. UEM Net [39] uses fully unsupervised tumor synthesis based on Markov Random Field enhancement, and MedMamba [40] incorporates synthetic ultrasound generated from CT to improve data diversity. Ref. [41] proposes an active learning framework for abdominal CT segmentation, where progressively refined pseudo-labels guide a 3D U-Net across training stages. Improvements from multimodal learning extend across modalities: the Swin Transformer approach of Pavarut et al. [14] adaptively weights available MRI and CT channels, and TotalSegmentator again leverages MRI+CT joint training to compensate for limited labeled data. In ultrasound, the DualInput pipeline of Alkhaldi et al. [42] uses pseudo-labeled public data to mitigate the shortage of expert annotations. These approaches rely on modality-specific characteristics (e.g., CT contrast or ultrasound texture), which may limit their transferability to non-contrast MRI. Additionally, methods based on synthetic data generation or pseudo-labeling are sensitive to inaccuracies in the generated annotations, which can propagate errors during training. Active learning strategies, while reducing annotation effort, still require iterative expert involvement, and multimodal approaches depend on the availability and proper alignment of heterogeneous datasets.

Outside the kidney segmentation context, ref. [43] develops a novel framework, SimTxtSeg, that leverages text cues to generate pseudo-labels, facilitating cross-modal fusion during model training. A Textual-to-Visual Cue Converter produces visual prompts from text prompts on medical images, enabling the Segment Anything Model to generate pseudo-masks. Then, a weakly supervised segmentation model with a text–vision attention module fuses text and image features to predict the segmentation mask. Another original approach to weakly supervised segmentation of gastrointestinal and T2-weighted MR images is presented in [44]. The authors propose a multi-level framework that trains multiple 2D U-Net networks in parallel. Each network is focused on a different level of detail, i.e., a bounding box, point set, or scribble. The pseudo-masks for each level are derived by applying hierarchical thresholds on gaze heatmaps. Lastly, ref. [45] addresses the problem of ambiguous anatomical boundaries and variability in expert-labeled annotations, which can introduce noise and affect the reliability of deep learning models. The authors introduce a method to generate probabilistic labels leveraging multi-rater annotations combined with a normalized so-called active–passive loss function to train in a noise-tolerant manner. The proposed approach is validated on MRI data of 17 knees for segmenting and detecting bone marrow lesions (BML). It demonstrates improvements of 14% in precision, 22% in recall, and 8% in Dice score compared to a binary cross-entropy loss function. Here, the main limitations arise from the need for auxiliary sources of supervision (e.g., text descriptions, gaze signals, or multi-rater annotations), which may not be readily available in standard clinical datasets. Additionally, the quality of the resulting pseudo-labels depends on the accuracy of intermediate representations (e.g., attention maps or heatmaps), and errors introduced at this stage may propagate to the final segmentation model. Furthermore, some methods are validated on relatively small or task-specific datasets, which may limit their generalizability to broader clinical scenarios.

Across semi-supervised, weakly supervised, and synthetic data methods, reported Dice scores for MRI-based kidney segmentation generally fall in the range of 0.90–0.93. CT-based approaches, by comparison, typically achieve values around 0.70–0.96, depending on anatomical structures, modality combinations, and supervision strength. These results indicate that reduced annotation methods can approach the performance of fully supervised models, especially when supported by cross-modality learning or high-quality synthetic augmentation.

## 3. Materials and Methods

### 3.1. Segmentation Model

All experiments described in this paper were conducted using the well-established 2D U-Net architecture for image segmentation. Our implementation follows the seminal encoder–decoder structure with skip connections [8]. It features a symmetric “U-shaped” design comprising an encoding path (contracting), a bottleneck, and a decoding path (expanding). The model is designed to capture multi-scale contextual information and enable precise pixel-wise localization.

The encoder consists of four downsampling blocks. Each block performs two sequential 3×3 convolutions, each followed by batch normalization and ReLU activation, to extract hierarchical features. Then, a 2×2 max-pooling operation is applied with stride 2, and padding 1 is applied for spatial downsampling, doubling the number of feature channels at each step. The sequence of feature channels in the encoder is 64, 128, 256, 512.

At the base of the network, the bottleneck layer processes the most downsampled features using a double convolution block, expanding the channel dimension to 1024. This layer serves as a bridge between the encoder and decoder, capturing high-level, abstract feature representations.

The decoder mirrors the encoder with four upsampling blocks. Each block begins with a 2×2 transposed convolution that halves the channel count and doubles the spatial resolution. The resulting feature map is then concatenated with the corresponding cropped feature map from the encoder path via a skip connection. This concatenation combines high-resolution contextual information from the encoder with the upsampled features, facilitating precise localization. A double convolution block follows to refine the merged features. The channel dimensions in the decoder are 512, 256, 128, and 64.

Generative AI tools were used for language editing of the manuscript, specifically Microsoft 365 Copilot and Grammarly (version 14.1304.0). All proposed corrections were thoroughly reviewed and verified by the authors, who take full responsibility for the final text of the manuscript. In addition, during the initial phase of this study, the OpenAI o3 model was used to facilitate the search for relevant literature. All references identified in this manner were independently checked by the authors to confirm that they originated from reliable sources and were not hallucinated.

### 3.2. Evaluation Metrics

#### 3.2.1. Dice Score

The Dice–Sørensen similarity coefficient (DSC) is an evaluation metric commonly used to evaluate the performance of biomedical image segmentation models. The DSC ranges from 0 to 1, where 1 indicates perfect overlap (identical sets) and 0 indicates no overlap. It is defined as follows:

(1) $$\mathrm{DSC}=\frac{2\cdot |A\cap B|}{\left|A|+|B\right|},$$

where A and B indicate the number of pixels in the predicted and ground-truth annotation regions. The smooth factor of 1 is added to the numerator and denominator as a way of avoiding division by zero and treating such cases as perfect predictions:

(2) $$\mathrm{DSC}=\frac{2\cdot |A\cap B|+1}{|A|+|B|+1}.$$

A division by zero occurs when the number of predicted kidney pixels is 0 and the number of pixels in the target label is also 0, indicating a perfect true-negative prediction. When either set A or set B is greater than zero while the other set remains zero, the Dice score is very low, nearly zero, as it would be without the smoothing factor. However, when both sets contain more than zero pixels, the impact of the smoothing factor is negligible. The number of pixels in such cases typically ranges from hundreds to thousands.

#### 3.2.2. Binary Cross-Entropy Loss

Binary cross-entropy ($\ell_{\mathrm{BCE}}$) is a loss function frequently employed in binary classification tasks to assess the disparity between predicted probabilities and actual binary labels. It quantifies how well a model’s predictions align with the true outcomes, imposing a heavier penalty on confident incorrect predictions:

(3) $$\ell_{\mathrm{BCE}}=-\frac{1}{N}\sum_{i=1}^{N}\left[y_{i}\cdot \log\left({\hat{y}}_{i}\right)+\left(1-y_{i}\right)\cdot \log\left(1-{\hat{y}}_{i}\right)\right],$$

where *N* is the total number of pixels, $y_{i}$ is the true binary label (0 or 1) for the *i*-th pixel, and ${\hat{y}}_{i}$ is the predicted probability that the *i*-th pixel belongs to class 1.

Since the model used in this study segments only the kidney mask, the problem can be simplified to a binary classification task, allowing the use of the BCE loss function. In this study, BCE was combined with Dice loss to more effectively address the sparsity of the loss function. Annotations for the kidney were often present in fewer than 40% of the slices in each scan; therefore, the final loss was calculated by summing the BCE and DSC losses, each weighted by the α and β factors as

(4) $$\ell =\alpha \cdot \ell_{\mathrm{BCE}}+\beta \cdot \ell_{\mathrm{DSC}}$$

### 3.3. Datasets

**Figure 1:**
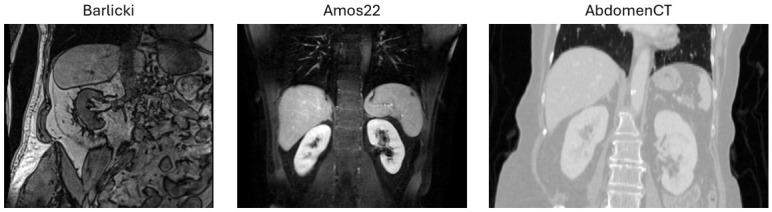
Example cross-sections from each of the datasets used in the study: on the left, the Barlicki dataset; in the middle, Amos22; and on the right, the AbdomenCT.

In this study, three datasets were used. The primary one was collected in the Barlicki Clinical Hospital in Lodz, Poland, in an independent study aiming to establish relationships between non-contrast MR image features and CKD-related renal tissue states [46]. The goal of the study was to investigate how different frugal learning techniques influence kidney segmentation models’ performance in an environment with a relatively small number of annotated data samples. For this task, the Barlicki dataset was used, alongside two additional image collections—AbdomenCT [47] and Amos22 MRI [48]—which were involved to facilitate frugal learning techniques such as transfer learning and data augmentation. Figure 1 shows example 2D slices from each dataset.

#### 3.3.1. Barlicki

The collection of 115 image volumes included in the study was partitioned into three subgroups based on the result of biopsy examination: 1—normally appearing (15% of subjects), 2—active lesions linked to ongoing inflammation (60%), and 3—chronic lesions characteristic of advanced CKD stage (25%). Scanning was performed on a 3T Magneton Vida unit (Siemens Healthcare GmbH, Erlangen, Germany). Each subject was imaged using the T1-weighted Dixon-VIBE technique, and thus had two mutually registered image data, the so-called in-phase (IP) and opposed-phase (OP) volumes, as is typically acquired in the Dixon method. By taking the sum of the volumes, one achieves a water-only (WO) image with fat being uniformly suppressed. Subtracting the opposed-phase volume from the in-phase volume brings, on the other hand, the fat-only (FO) image. For the models developed in this study, only the OP scans were used due to their high contrast-to-noise ratio and good separation between the kidney and other organs. The images were acquired with the following scanning protocol: TR = 4 ms, TE1 = 1.26 ms, TE2 = 2.4 ms, TA = 14.78; voxel size: reconstruction—1.4×1.4×2.0 mm, acquisition—1.56×1.41×4.00 mm; FOV = 450 mm; number of slices—64. Each kidney (left and right) on the majority of scans was imaged separately in two independent sequences. Thanks to this separation, it was possible to align the scanning plane with the quasi-coronal orientation of each individual organ, which is crucial to properly assess the renal tissue in 2D cross-sections. The examination protocol was approved by the Bioethical Committee associated with the Medical University of Lodz (decision RNN/206/20/KE, dated 8 September 2020). The dataset was partially annotated by an expert in medical image analysis. A total of 65 ground-truth segmentations were available for analysis. This annotated fraction contained the same proportions of patients from each category as the full dataset. The remaining images were left unlabeled, as we opted to employ a frugal learning methodology to develop the final segmentation models.

#### 3.3.2. Amos22 MRI

The Abdominal Multi-Organ Segmentation (AMOS) dataset was introduced alongside the publication [48] to advance the field of abdominal organ segmentation in medical imaging. This dataset addresses the limitations of existing resources by offering a large-scale, diverse, and clinically relevant benchmark for evaluating segmentation algorithms. It comprises 500 CT scans and 100 MRI scans collected between 2018 and 2021 from two hospitals in Shenzhen, China, using eight different scanners. The scans were obtained from patients with various abdominal conditions. Annotation preparation followed a semi-automatic workflow, beginning with coarse labeling using pretrained models (e.g., 3D-UNet, VNet) followed by refinements from five junior radiologists and validation by three senior specialists. Annotations were created for 15 organs, including the left and right kidneys. In this paper, only the MRI scans were used with only kidney annotations.

In addition to releasing the dataset, the authors also benchmarked several widely used deep learning models, including the abovementioned U-Net and UNETR, as well as V-Net [49], Co-Tr [50], nnFormer [51], and Swin-UNETR [52]. In their evaluation, the original U-Net model achieved the highest validation and test Dice scores despite being the smallest and oldest model among the tested architectures. This result supports the selection of U-Net as the model of choice in this study.

#### 3.3.3. AbdomenCT-1K

The AbdomenCT-1K dataset, introduced in [47], aims to address the lack of diversity in existing datasets, which are often restricted to single-center or single-disease cases. This dataset encompasses multi-center, multi-phase, multi-vendor, and multi-disease cases, with annotations provided for four major abdominal organs: liver, kidneys, spleen, and pancreas. It includes 1112 CT scans collected from 12 medical centers; however, the authors did not disclose details regarding the annotation process or the locations where the scans were prepared. In the current work, the entire dataset was utilized, but with annotations limited to kidneys.

### 3.4. Experimental Setup

**Figure 2:**
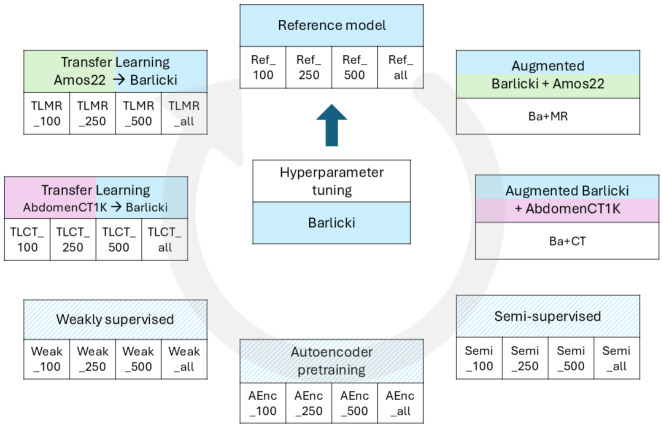
Schematic representation of the experimental workflow. The study starts with the central block representing hyperparameter tuning on the Barlicki dataset. Next, using each of the evaluated methods, the base models are trained. Each block header represents the base model of the given method, and it is color-coded with respect to the dataset involved: Barlicki (target dataset of this study)—blue, Amos22 (MRI auxiliary dataset)—green, AbdomenCT-1K (CT auxiliary dataset)—magenta. Base model prefixes correspond to the learning method: *Ref*—reference, *Ba+MR/CT*—data augmentation, *Semi*—semi-supervised learning, *AEnc*—autoencoder pretraining, *Weak*—weak supervision, *TLCT/TLMR*—transfer learning. After training, each base model is fine-tuned using the same subsets of the Barlicki dataset across all methods; the bottom rows of each block show the number of samples from the Barlicki dataset used to fine-tune the base models. Finally, the performance of the fine-tuned models is compared.

The experiments aimed to evaluate which learning method yields the best results in kidney segmentation using a limited number of annotated non-contrast T1-weighted images. The workflow starts with initial training the aim of which is to determine the optimal hyperparameters used throughout the study. Secondly, a baseline model is constructed in a standard manner using only the Barlicki dataset. This baseline model serves as a reference for comparing with the 7 considered frugal learning techniques. Next, for each method, the base model is trained, and then each base model is fine-tuned using the same subsets of the Barlicki dataset. The overall procedure is illustrated in Figure 2. In the figure, the datasets included in each training experiment are color-coded according to the block representing the given training setup. A more detailed description of the experimental configurations is provided below.

For training, the Barlicki dataset is split into three subsets: the training, validation, and test sets. The samples for the test set are handpicked to include all three conditions analyzed in the study (normally appearing scans, active lesions, chronic lesions). In total, 5 scans are selected, representing approximately 8% of the entire Barlicki dataset. The training and validation sets are created by applying a random 80/20 split to the remaining data after excluding the test set. The sampling is performed without regard to slice order, meaning slices from a given scan may appear in both the training and validation sets. The same splits are used across all training unless stated otherwise.

#### 3.4.1. Base Training

Prior to evaluating the frugal learning methods, an extensive hyperparameter optimization study is conducted using all annotated samples from the Barlicki dataset. Approximately 80 training runs are performed to identify the optimal values for each hyperparameter.

To ensure reproducibility of the training process, all random number generators are initialized with fixed seeds. Furthermore, the cuDNN backend is configured to operate deterministically, and is consistently maintained during the evaluation of all tested frugal learning methods. The final set of hyperparameters is applied consistently across all training runs.

Subsequently, reference models are developed using only the Barlicki dataset to provide a baseline for comparison with the tested training methods. A series of the following standard models is developed: one trained on the full Barlicki dataset and three trained on the same subsets as used for fine-tuning other methods’ base models. These reference models are trained from randomly initialized weights. Eventually, a series of base models is developed, one for every examined frugal learning method (cf. Table 1).

#### 3.4.2. Fine-Tuning Process

Direct comparison of the base models is not feasible due to the differences in the training procedures. Some methods utilize annotated samples from the Barlicki dataset to train the base model, whereas others, such as autoencoder-based pretraining, do not rely on any labeled data. In the latter case, the convolutional filters capture a general representation of data rather than kidney segmentation-specific features. Thus, to ensure a fair and meaningful comparison, each base model is fine-tuned on the same subsets of the Barlicki dataset prior to performance evaluation.

During fine-tuning, the same hyperparameter configuration as in the initial training is retained, with one modification: the learning rate is reduced from $10^{-3}$ to $10^{-4}$. This adjustment reflects the objective of fine-tuning, which is to adapt the pretrained weights in the direction that gives the best results on the entire Barlicki dataset rather than to relearn or overfit to its subset. The fine-tuning process is conducted for four scenarios, depending on the number of patient scans and slices included in the final training dataset, sampled from the training set:

1. 100 slices from 2 scans;
2. 250 slices from 4 scans;
3. 500 slices from 8 scans;
4. Entire Barlicki dataset—2622 slices from 60 scans.

For each fine-tuning experiment, the full Barlicki validation set is used.

In Figure 2, each fine-tuned model is named according to its pretraining scheme code (cf. Table 1) and the number of slices from the Barlicki dataset employed in the fine-tuning phase. The fine-tuned models are evaluated using scans from 5 subjects, representing approximately 8% of the entire Barlicki dataset and encompassing all three conditions analyzed in the study. Expanding the test set further would significantly reduce the number of data available for model development. Importantly, none of the test samples is included in the training or validation datasets across any of the experiments.

#### 3.4.3. Data Augmentation

The first frugal learning approach involves combining the training subset of the Barlicki dataset with one of the two additional datasets. We refer to this approach as data augmentation, as it enhances the training set by increasing its diversity and introducing new samples, albeit sourced from a different dataset than the target images. Two experiments are conducted using this method: in the first experiment, the Barlicki training set is augmented with data from the AbdomenCT dataset; in the second, it is augmented with data from the Amos22 dataset. In both cases, an equal number of slices (2622 in total) is selected from the additional dataset to match the size of the Barlicki training set. This balanced augmentation ensures that neither dataset disproportionately influences the training distribution, thereby reducing potential bias toward the characteristics of a single dataset. To increase diversity in the training samples, slices are selected from AbdomenCT at intervals of every fourth slice, whereas slices from Amos22 are sampled sequentially with a step size of one. During the training with each method, all samples from the Barlicki training set are used. That is why no fine-tuning is performed in this case.

This augmentation-based approach is expected to improve the model’s robustness in detecting kidney characteristics, such as edges and textures, across varied imaging conditions. By incorporating diverse yet related samples, the model should better capture the kidney’s anatomical invariants and thus potentially outperform training on the Barlicki dataset alone, particularly for scans with suboptimal quality or with some anatomical variations.

#### 3.4.4. Semi-Supervised Learning

In this approach, the original annotations used for training are replaced with predictions generated by the baseline U-Net model. These pseudo-labels are created for all 110 scans, including those without manual kidney masks, which were not used during the training of the base model. In other words, the entire Barlicki dataset, excluding the test set, is utilized for pseudo-label generation.

This method aims to enhance the model’s understanding of kidney characteristics by exposing it to a larger set of pseudo-annotated samples. By leveraging this expanded dataset, the U-Net’s convolutional filters are expected to more effectively capture anatomical features, such as kidney boundaries and textures. This, in turn, should improve the model’s robustness to variations in scan quality and orientation compared to training solely on ground-truth annotations.

Note, in this study, pseudo-labels are generated in two different contexts: the first is a semi-supervised learning scheme, as described here; the second is a weakly supervised setting, where activation maps derived from a classification model are converted into approximate segmentation masks. Although both approaches rely on automatically generated labels, they differ in their underlying methodology and supervision assumptions.

#### 3.4.5. Autoencoder Pretraining

**Figure 3:**
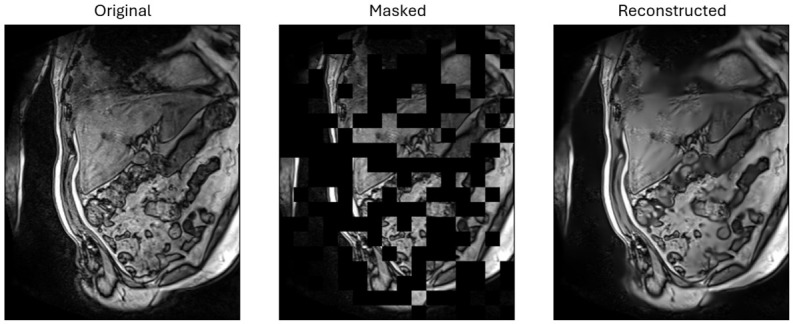
Illustration of the masked autoencoder (MAE) pretraining process for the AEnc base model using the Barlicki dataset. (**Left**): An original MRI cross-section. (**Middle**): The randomly masked input image presented to the network during training. (**Right**): The reconstructed output generated by the trained U-Net architecture.

The U-Net architecture, characterized by its encoder–decoder structure, is well-suited for pretraining as an autoencoder. In this work, a masked autoencoder (MAE) [53] approach is adopted. The input images are divided into a grid of 256 equally sized patches, 16 patches in width and 16 in height. During each training step, 50% of these patches are randomly masked. The task of a network is to reconstruct the missing regions based on the unmasked patches. This process is shown in Figure 3.

The pretraining process utilizes base hyperparameters tailored to this task. The mean squared error (MSE) loss function is used to measure the difference between the reconstructed and original slices. In this training, all 110 scans of the Barlicki dataset are used—the entire dataset except the test subset. Pretraining in this manner allows the model to effectively learn the underlying characteristics of the input data. The convolutional filters are expected to extract both low-level features, such as edges and textures, and high-level semantic structures. In theory, fine-tuning a pretrained model for the segmentation task should be relatively straightforward, as the model already possesses a well-developed representation of the data. The fine-tuning process in this case should primarily involve learning to identify and segment the specific patterns corresponding to kidney structures.

During fine-tuning, two strategies are evaluated: in the first approach, only the decoder of the network is trained while the encoder remains frozen; in the second approach, the entire model is fine-tuned. For both methods, the final layer of the U-Net model is reinitialized with randomly assigned weights. The first method gives better results, and, in following results, only this approach is presented.

#### 3.4.6. Weak Labels

In this weak supervision experiment, we use activation maps generated by Gradient-Weighted Class Activation Mapping (Grad-CAM) [54] as segmentation masks (pseudo-labels) for the base segmentation model training. Activation maps are often used in explainable AI to highlight the area in the input image that convinced the classification model to predict the given label. Since well-trained classification models should base their prediction on the presence of predicted objects in the image, an activation map should highlight the discussed object.

To generate pseudo-annotations, the ResNet18 [55] model is first trained to classify whether a kidney is present in a 2D slice of a scan. The model is initialized using the TorchVision model zoo [56] with weights pretrained on ImageNet [57]. Prior to training, the classifier layer of the ResNet18 is replaced with a custom layer containing two output neurons: one representing the probability of kidney presence and the other representing its absence.

The entire Barlicki dataset is utilized for training this method’s base model, with classification labels derived from kidney segmentation masks. Specifically, if the ground truth contains a kidney, the label indicating kidney presence is assigned; otherwise, the label indicating kidney absence is used.

#### 3.4.7. Transfer Learning

To complete the comparative analysis, the U-Net model is pretrained using the Amos22 and AbdomenCT-1K datasets. Pretraining on a larger, external dataset is a widely adopted strategy for enhancing model performance. This approach leverages the ability of convolutional filters to learn general structural patterns within the pretraining data, particularly when the pretraining dataset shares similarities with the fine-tuning dataset. During fine-tuning, these filters can be further refined to capture dataset-specific features. In this study, all three datasets focus on abdominal imaging, albeit with variations in imaging modalities, scan regions, and angles. Despite these differences, all datasets include annotations for the same target organ—the kidneys. This shared annotation focus suggests that initializing the training process on the Barlicki dataset using weights derived from pretraining on the other two datasets is likely to improve the final model’s performance.

For the pretrained models, the same hyperparameters are employed as in the other training procedures. Pretraining on the Amos22 dataset utilizes all available MRI samples. In the case of the AbdomenCT-1K dataset, due to its large size, every fourth slice from each scan is selected for training, as the full dataset exceeds the available resources of the training machine. Both models are exclusively trained for kidney segmentation, with other anatomical classes excluded from consideration.

Following pretraining, the same fine-tuning procedure is applied as in the other methods. Notably, no layer-freezing strategy is implemented during the fine-tuning process.

## 4. Results

The results are presented in the following way: the first sub-section reports the performance on the validation set, the second on the test set, and, in the end, there is a visual review of the results.

Both evaluations are important for assessing the proposed frugal learning methods. The validation set reflects typical data from the Barlicki dataset and is used to measure overall model performance. The test set, by contrast, was manually selected to include various stages of kidney abnormalities and is used to evaluate performance on difficult edge cases. When the same model was trained or fine-tuned multiple times with small changes, test results varied by a few percentage points, while validation results stayed more consistent. Therefore, validation metrics are at least as important—and often more useful—than test results when comparing methods. Their stability makes them a better measure of average model performance on the Barlicki dataset. The same validation set is used for all fine-tuning experiments in this section.

Given that the Barlicki dataset contains only 65 annotated kidney scans, it was not feasible to create multiple independent test sets. Doing so would have significantly reduced the number of data available for training. Therefore, performance comparisons were restricted to these two dataset splits: the validation set and the test set.

To assess whether the observed performance was affected by overfitting, the training and validation loss curves were monitored throughout all the training and fine-tuning stages. The curves exhibited consistent trends without divergence, indicating a stable generalization behavior. In addition, the final models were evaluated on an independent test set. The test set metrics were slightly lower than those obtained during training and validation; however, the decrease remained proportional and consistent in all evaluated methods. Importantly, no systematic performance degradation was observed for any specific CKD subgroup. Although the test set was limited in size due to the limited number of annotated samples, it provided an additional indication that the proposed frugal learning approaches did not overfit to the training data.

### 4.1. Validation Set Results

**Figure 4:**
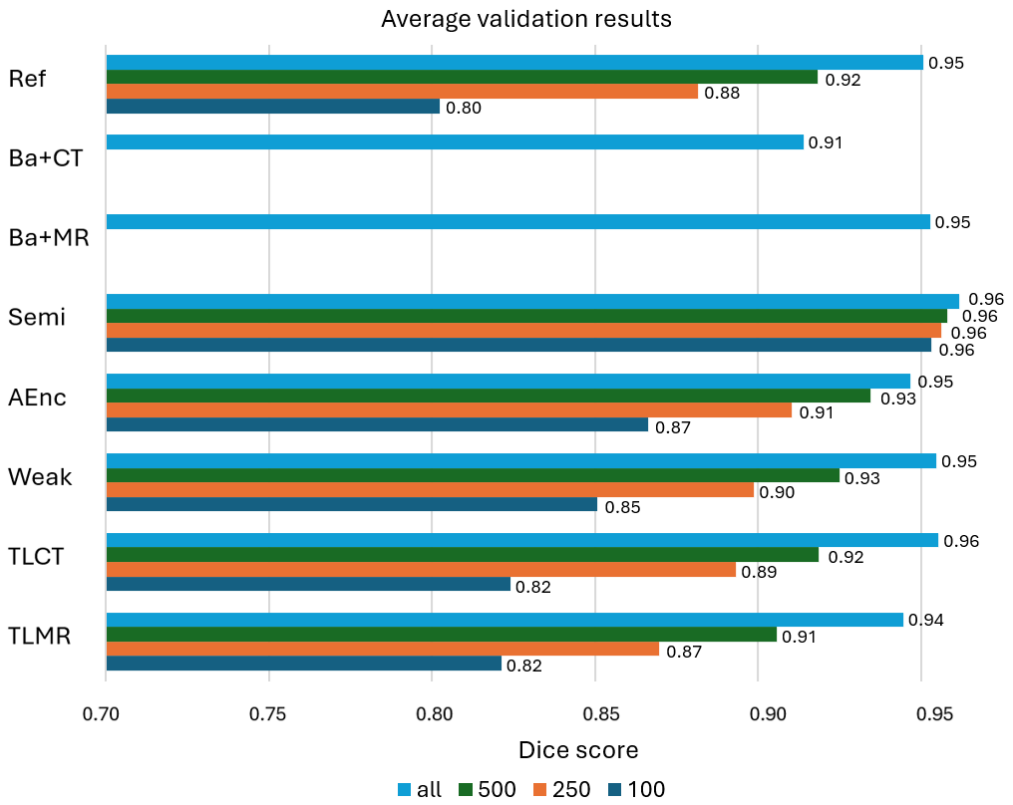
Comparison of the maximum validation Dice scores, obtained during fine-tuning by models pretrained with different frugal learning techniques and fine-tuned on identical subsets of the training data (100, 250, 500, and all available MRI slices from the Barlicki dataset). For each configuration, the reported value is the Dice score averaged over all validation samples at the epoch where this average was highest. All scores were evaluated on the same full validation set to maintain consistency across experiments. Abbreviations: *Ref*—reference, *Ba+MR/CT*—data augmentation, *Semi*—semi-supervised learning, *AEnc*—autoencoder pretraining, *Weak*—weak supervision, *TLCT/TLMR*—transfer learning.

When examining validation metrics, the semi-supervised, autoencoder pretraining, and weakly supervised techniques demonstrated clear improvements over the base model. Other techniques boost model performance when training or fine-tuning on smaller data splits. All results are presented in Figure 4.

The semi-supervised learning approach achieved the highest Dice score among all fine-tuned models: 0.9616. This indicates that expanding the training data using model-generated pseudo-labels, followed by fine-tuning on manually annotated data, yields better segmentation performance than relying on the limited manual annotations alone.

The pretraining-as-autoencoder method also performed strongly, particularly considering its base model was trained without any manual annotations. It showed notable robustness when fine-tuned on smaller subsets of data. For instance, when fine-tuned on only approximately one and a half scans (about 100 samples), it achieved a Dice score of 0.8663, which was the best result excluding that of the semi-supervised learning approach, which had access to the full dataset during pretraining. Excluding it, the autoencoder pretraining also achieved the top performance for the 250- and 500-sample fine-tuning experiments. This performance stability may be attributed to the method’s reliance on a pretrained latent space representation and the partial network retraining strategy.

The transfer learning experiments produced results comparable to the base model, indicating limited benefit in this specific configuration. Finally, the experiments involving merged datasets showed mixed outcomes: the model fine-tuned with the merged Abdomen dataset underperformed relative to the base model, whereas fine-tuning with the merged Amos dataset yielded a slight improvement.

### 4.2. Test Set Results

When trained on the entire Barlicki dataset, the base model achieved the best overall performance on the test set. The number of data available for training was sufficient for effective convergence, and applying additional frugal learning techniques did not yield further improvement, although individual methods outperformed the base model on specific fine-tuning sets.

**Figure 5:**
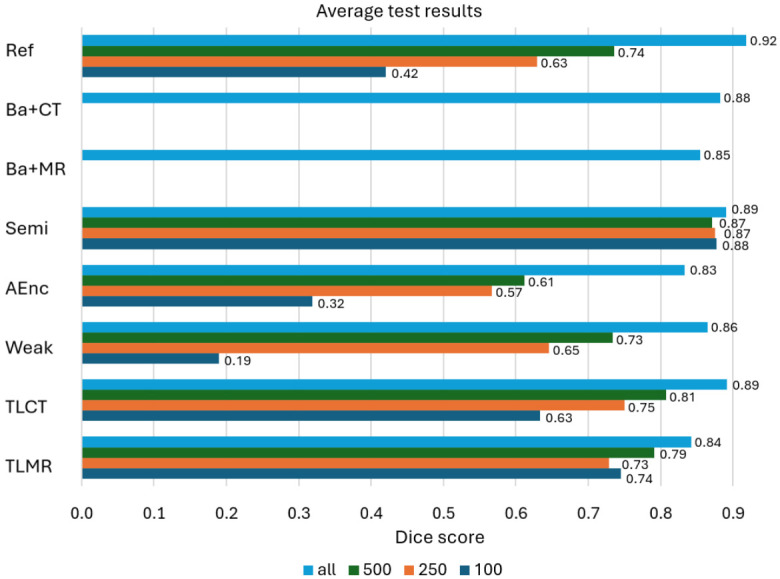
Dice scores obtained on the test set by all evaluated models. Models were pretrained with different frugal learning techniques (*Ref*—reference, *Ba+MR/CT*—data augmentation, *Semi*—semi-supervised learning, *AEnc*—autoencoder pretraining, *Weak*—weak supervision, *TLCT/TLMR*—transfer learning) and fine-tuned on identical subsets of the training data (100, 250, 500, and all available MRI slices from the Barlicki dataset). The metrics were calculated using the same 5 manually selected test scans, chosen to capture the full range of kidney abnormalities found in the Barlicki dataset, and averaged over these 5 scans for each configuration.

When examining the fine-tuning experiments on smaller subsets of the Barlicki dataset, the semi-supervised learning and transfer learning methods demonstrated superior performance. These approaches noticeably outperformed models initialized with random weights, indicating their effectiveness in low-data regimes. The semi-supervised learning method benefited from leveraging model-generated labels to augment limited training data, while transfer learning capitalized on pre-learned feature representations, resulting in more accurate segmentation despite the constrained dataset size. All the results are presented in Figure 5.

### 4.3. Visual Results

**Figure 6:**
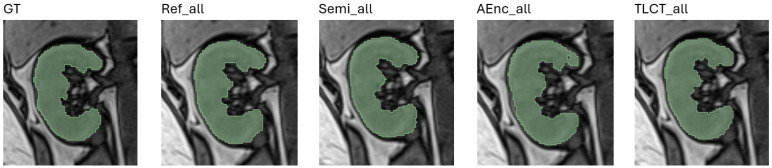
Ground-truth (GT) and predicted segmentation masks (green overlay) for test scan PT91/Opp-R, generated by models fine-tuned on the entire Barlicki dataset. Models included in the comparison (from left to right, skipping left-most GT): *Ref*—reference, *Semi*—semi-supervised learning, *AEnc*—autoencoder pretraining, *TLCT*—transfer learning using AbdomenCT dataset.

To provide a qualitative assessment of segmentation performance, a visual review was conducted. Figure 6 presents the cross-section from one representative test scan with segmentation masks generated by the best models. This scan was selected because it exhibits no structural deformations and closely resembles the majority of training samples from the Barlicki dataset.

**Figure 7:**
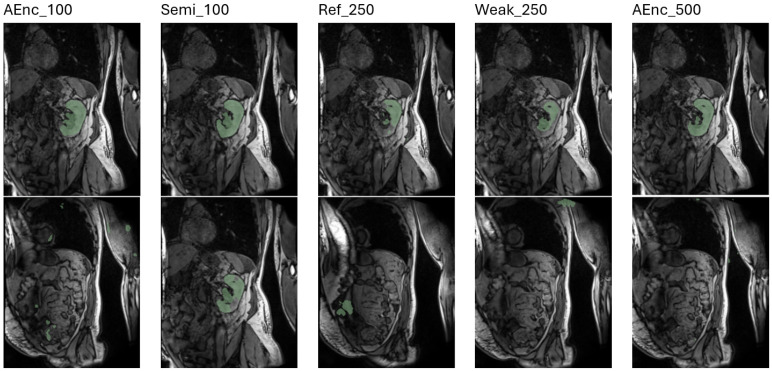
Showcase of test results (green overlay) predicted by models fine-tuned on smaller amounts of samples. Top and bottom rows show different test slices, illustrating a representative good prediction and a typical error, respectively, for each method. Base models included in the comparison: *AEnc*—autoencoder pretraining, *Semi*—semi-supervised learning, *Ref*—reference, *Weak*—weak supervision. Model suffixes denote fine-tuning on 100, 250, or 500 slices from the Barlicki dataset.

Figure 7 shows cross-sections of models fine-tuned on smaller numbers of data. The predictions generated by different frugal learning methods look better than the predictions of the base model. The base model does not have as many false positives, but the kidney masks generated by it are more under-segmented.

**Figure 8:**
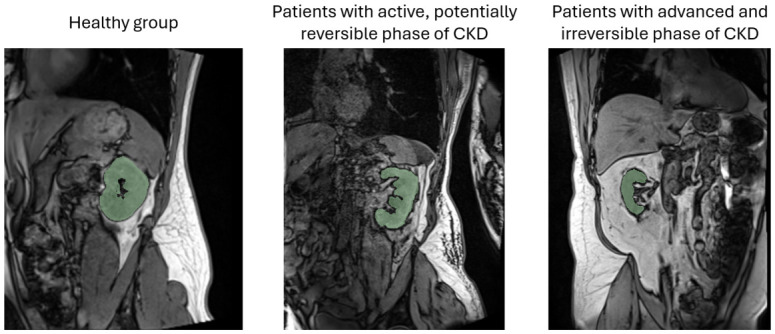
Predicted segmentation masks (green overlay) by the model trained in the semi-supervised manner (*Semi_all*) for 3 groups of test patients from the Barlicki dataset, with the groups representing different stages of chronic kidney disease (CKD).

Different methods exhibited distinct error patterns. Specifically, the autoencoder pretraining tended to produce scattered noise; however, the resulting kidney masks reliably covered the full renal parenchyma, with only moderate over-segmentation in the pelvic region. This behavior was observed even when fine-tuning was performed using only 100 training samples. In contrast, the weakly supervised model showed imprecise mask boundaries but yielded the fewest false-positive predictions. Overall, the annotations produced by the semi-supervised method achieved the highest quality; however, its training requires a strong base model. Figure 8 presents predictions for all three patient subgroups from the Barlicki dataset. Model performance was satisfactory across all three cases, despite the uneven representation of these groups in the dataset.

## 5. Discussion

To provide appropriate context for the frugal learning results, the performance of the base reference model was first compared with the state-of-the-art methods reported in the literature. Table 2 summarizes a comparison of MRI-based kidney segmentation studies, limited to contributions explicitly addressing the kidney segmentation task or at least including the kidney within a broader multi-organ segmentation framework. As can be observed, the reported Dice scores ranged from 0.839 to 0.941, reflecting substantial variability across architectures, imaging protocols, and dataset sizes. Methods trained on relatively small and pathology-specific cohorts—such as that in [12]—reported Dice values up to 0.94 when using T2-weighted or FSE T2-weighted sequences. In contrast, large-scale or multi-organ approaches exhibited lower Dice scores, highlighting the increased complexity of these settings. Our model (*Ref_all*), trained on Dixon-based T1-weighted MRI from 60 CKD patients, achieved a Dice score of 0.920, which is comparable to prior kidney-specific approaches. This performance was achieved while relying on a clinically common acquisition protocol and a moderately sized cohort. Notably, T1-weighted Dixon-based imaging is underrepresented among existing studies. The only work that also focused on this technique is [6], where the achieved DSC was lower than in our results.

The presented results suggest that frugal learning techniques can achieve performance comparable to conventional supervised training while reducing dependence on manual annotations. The differing performance profiles of the evaluated approaches can be explained by their underlying learning mechanisms. Among the evaluated methods, the semi-supervised approach performed best overall, achieving the highest validation Dice score and top performance in three out of five test cases. The autoencoder pretraining method showed the strongest performance in very-low-data settings, producing plausible kidney masks even with minimal fine-tuning; nonetheless, scattered noise artifacts were occasionally observed. This behavior may stem from latent representations learned during self-supervised pretraining, which do not always capture the anatomical characteristics most relevant for downstream segmentation. Weak-label learning yielded intermediate performance and showed potential in annotation-constrained scenarios but tended toward over-segmentation due to the limited precision of the supervisory signal. More traditional strategies, such as dataset merging or pretraining on larger datasets, also improved performance when fine-tuned on small subsets. However, their effectiveness may be constrained by differences between source and target data domains. Across methods, common challenges include boundary localization errors and occasional false positives, highlighting opportunities for further refinement. The semi-supervised approach additionally depends on a sufficiently accurate initial model for pseudo-label generation. Overall, these findings indicate that different frugal learning strategies offer complementary advantages depending on annotation availability and deployment constraints. A major strength of the present study is the systematic evaluation of multiple frugal learning strategies under identical experimental conditions. This design enables a direct and methodologically consistent comparison across annotation-constrained scenarios.

It is also instructive to place the designed models in the context of alternative frugal learning-based approaches applied to kidney-related segmentation tasks across a variety of imaging modalities (see Table 3). Despite differences in tasks, datasets, and acquisition protocols, the Dice scores of our three best variants are comparable to those of prior methods using data augmentation or active, transfer, or semi-supervised learning. Notably, some studies relied on contrast-enhanced CT or MRI, which offers clearer tissue delineation than the non-contrast-enhanced Dixon-VIBE MRI used here. Additionally, our focus on chronic kidney disease introduced significant anatomical variability due to renal atrophy, further complicating segmentation. Comparisons remain limited by heterogeneity in imaging modalities, task definitions, datasets, and evaluation protocols, making the table primarily contextual rather than a definitive benchmark.

Several limitations should be acknowledged: First, the target dataset contained only 65 annotated scans, which restricted the size of the independent test cohort and prevented more extensive external validation. Second, the Barlicki dataset originated from a single clinical center and a specific MRI protocol, which may limit generalizability to other acquisition settings. Third, some frugal learning approaches depend on the quality of pseudo-labels, weak annotations, or external datasets, meaning that errors present in these auxiliary sources can propagate to the segmentation model. Finally, the comparisons with previously published studies summarized in Table 2 and Table 3 should be interpreted cautiously because of differences in patient populations, imaging protocols, dataset sizes, and evaluation methodologies.

From a clinical perspective, the proposed framework has several practical implications: First, the ability to train segmentation models under limited annotation availability is particularly relevant in routine clinical environments, where expert labeling is costly and time-consuming. It indicates that automated segmentation tools may be developed even in centers with relatively small datasets. In such settings, assembling large manually annotated MRI repositories is often impractical because expert delineation is costly and time-consuming. The evaluated frugal learning approaches leverage unlabeled examinations, weak annotations, or external datasets to compensate for this limitation, thereby reducing the amount of manual annotation required for model development. As a result, institutions with limited imaging resources may still be able to implement automated kidney segmentation workflows and develop quantitative MRI biomarkers.

One immediate application is the automatic estimation of total kidney volume (TKV), a well-established biomarker for assessing kidney size and monitoring chronic kidney disease progression. By providing consistent and reproducible kidney masks, the proposed models can facilitate large-scale and longitudinal volumetric analysis. Furthermore, the framework can be extended to more detailed anatomical structures, such as the renal cortex and medulla. In this scenario, additional biomarkers—including cortical volume, cortical thickness, or cortex-to-medulla ratios—could be derived, offering more sensitive indicators of disease progression than global kidney volume alone.

Beyond volumetric measurements, automated segmentation may support clinical workflows by reducing the time required for manual delineation, improving reproducibility across readers, and enabling more standardized reporting. It may also facilitate retrospective studies and population-level analyses, where consistent segmentation across large datasets is required. Finally, robust segmentation under non-contrast MRI conditions could be particularly valuable for patients with impaired renal function, for whom contrast-enhanced imaging is contraindicated. This would thereby expand access to quantitative imaging biomarkers in this population.

## 6. Conclusions

In this work, we investigated the applicability of frugal learning techniques for kidney segmentation in non-contrast T1-weighted MRI under conditions with limited annotated data. The presented results indicate that several data-efficient strategies—particularly semi-supervised learning and data augmentation—have the potential to improve segmentation performance compared to standard supervised training when annotations are scarce.

To the best of our knowledge, this study is the first to systematically compare a diverse set of frugal learning methodologies on the same dataset under consistent evaluation conditions, enabling a fair and methodologically sound assessment. Taken together, these findings indicate that the observed performance differences relative to prior work are largely attributable to variations in imaging protocols and patient populations rather than methodological shortcomings. These results support further investigation of frugal learning approaches for MRI-based kidney segmentation in CKD cohorts.

## Figures and Tables

**Table 1:** Summary of the evaluated frugal learning methods and the specific data configurations used to train the base models. The table details the model prefixes assigned to each method with the exact number of scans and annotation types. The Barlicki dataset serves as the target dataset for this study, while Amos22 and AbdomenCT-1K act as auxiliary datasets (MRI and CT, respectively), utilized for data augmentation and transfer learning.

Learning Method		Model Prefix	Data Involved in Training the Base Model
Reference		*Ref*	60 annotated scans from Barlicki dataset
Data augmentation	with CT images	*Ba+CT*	60 scans from Barlicki + 1112 from AbdomenCT-1K
	with MR images	*Ba+MR*	60 scans from Barlicki + 100 from Amos
Semi-supervised		*Semi*	60 annotated scans from Barlicki to train the base model, and 110 scans with pseudo-annotations
Autoencoder pretraining		*AEnc*	110 scans from Barlicki dataset, all w/o annotations
Weak labels		*Weak*	60 annotated scans from Barlicki with just classification labels for kidney presence
Transfer learning	from CT images	*TLCT*	1112 scans from AbdomenCT-1K
	from MR images	*TLMR*	100 scans from Amos22

**Table 2:** Performance of the trained reference model (*Ref_all*) in comparison to other studies on kidney segmentation in non-contrast MR images.

Authors	Architecture	No. Subjects	MRI Sequence	DSC
Inoue et al. [6]	3D convolutional network	170 (70 renal disease)	Dixon-based T1-weighted	0.902
Al-Salman and Cevik [10]	Functionally guided U-Net	100 (50 CKD patients) ^1^	T2-weighted	0.940
Das et al. [12]	Two connected U-Nets with ASPP blocks	40 ADPKD patients	FSE T2-weighted	0.941
Sharaby et al. [36]	Cycle-consistent GAN	34 graft kidneys	BOLD	0.920
Zhang et al. [37]	2D U-Net	20 ^2^	SPIR T2-weighted	0.912, 0.890 ^4^
D’Antonoli et al. [38]	nnU-Net (TotalSegmentator)	616 ^3^	Multiparametric	0.839
Ours *Ref_all*	2D U-Net	60 CKD patients	Dixon-based T1-weighted	0.920

^1^ The model was developed on the combined MR/CT data, with 12,446 CT images included. ^2^ Images from 16 subjects were used to synthesize additional training data. Dice scores were obtained for the remaining 4 patients. ^3^ Right and left kidney scores, respectively. ^4^ The model was developed on the combined MR/CT data, with 527 CT images included. Presented Dice score was obtained in multi-organ study for test MRI images.

**Table 3:** Performance comparison of our three best-performing model variants with prior frugal learning-based kidney-related segmentation studies. DSC—Dice similarity coefficient.

Authors	Learning Technique	Task	Imaging Modality	DSC
Gregory et al. [19]	Active learning	Kidney cysts segmentation	MRI	0.902
Gregory et al. [20]			CE-CT	0.850
Kim et al. [41]		Kidney segmentation	CT	0.963
Pavarut et al. [14]	Cross-modality (MRI + CT) learning	Kidney tumor segmentation	MRI	0.730–0.786
D’Antonoli [38]		Multi-organ segmentation	Multiparametric MRI	0.839
Guo et al. [34]	Data augmentation by image synthesis	Kidney segmentation	CE-MRI	0.930
Sharaby et al. [36]			BOLD MRI	0.920
Zhang et al. [37]		Multi-organ segmentation	SPIR MRI	0.890–0.921
Wang et al. [40]			US	0.979
Li et al. [39]	Unsupervised learning	Kidney tumor and kidney segmentation	CT	0.952–0.972
Alkhaldi [42]	Semi-supervised	Kidney segmentation	US	0.890
Our *Semi_all* ^1^	Semi-supervised	Kidney segmentation	Dixon-Vibe MRI	0.890
Our *TLCT_all* ^2^	Transfer learning			0.890
Our *BA+MR* ^3^	Data augmentation			0.880

^1^ Base model trained in semi-supervised mode. Fine-tuning on entire Barlicki dataset. ^2^ Base model trained using transfer learning from AbdomenCT-1K dataset. Fine-tuning on entire Barlicki dataset. ^3^ Model trained on the Barlicki dataset augmented with Amos22 MRI dataset.

## Data Availability

The Barlicki dataset used in this study is unavailable due to privacy restrictions; the consent signed by the included subjects did not cover public access to the examination results. Additional datasets employed in the experiments (AMOS and AbdomenCT-1K) are publicly available, and the appropriate sources have been explicitly acknowledged in the manuscript.
